# Shared neurobiological changes in individuals with Crohn’s disease and major depressive disorder

**DOI:** 10.1038/s43856-025-01117-w

**Published:** 2025-09-17

**Authors:** Hanna A. Hartmann, Marja L. Berthold, Shukti Ramkiran, Lukas Bündgens, Julius W. Jaeger, Jana Hagen, Maria Backhaus, Maria Collée, Gereon J. Schnellbächer, Tanja Veselinović, N. Jon Shah, Kai M. Schneider, Ravichandran Rajkumar, Irene Neuner

**Affiliations:** 1 Department of Psychiatry, Psychotherapy and Psychosomatics, RWTH Aachen University, Aachen, Germany; 2 Institute of Neuroscience and Medicine 4, INM-4, Forschungszentrum Jülich, Jülich, Germany; 3 Center for Computational Life Science, RWTH Aachen University, Aachen, Germany; 4 Department of Gastroenterology, Metabolic Diseases and Internal Intensive Care Medicine, Uniklinik RWTH Aachen, Aachen, Germany; 5 JARA-BRAIN, Aachen, Germany; 6 Department of Neurology, RWTH Aachen University, Aachen, Germany; 7 Institute of Neuroscience and Medicine 11, INM-11, Forschungszentrum Jülich, Jülich, Germany; 8 Department of Medicine 1, University Hospital Carl Gustav Carus Dresden, Technische Universität (TU), Dresden, Germany; 9 Else Kroener Fresenius Center for Digital Health, Medical Faculty Carl Gustav Carus, TUD Dresden University of Technology, Dresden, Germany; 10 Center for Regenerative Therapies Dresden (CRTD), Technische Universität (TU), Dresden, Germany

**Keywords:** Neuroscience, Functional magnetic resonance imaging

## Abstract

**Background:**

Emerging evidence highlights the profound impact of the central nervous system on the gut. This is particularly evident in inflammatory bowel disease, where psychological stress has been shown to modulate the inflammatory response and precede disease flares. The neuropsychological correlates of the brain’s involvement in Crohn’s disease (CD), as well as its overlapping neurobiological pathways with major depressive disorder (MDD), remain poorly defined. This study aims to delineate these shared mechanisms using ultra-high-field neuroimaging.

**Methods:**

Resting-state functional magnetic resonance imaging was conducted on 13 CD patients (age range: 20–41 years; 9 males), 13 age-matched MDD patients (20–42 years; 9 males), and 13 healthy controls (HC) (19–42 years; 9 males) using a 7 Tesla MR scanner. Assessments for symptom severity included the Beck Depression Inventory-II (BDI-II) for depression and the Gastrointestinal Symptom Rating Scale (GSRS) for CD.

**Results:**

Significant differences in BDI-II scores are observed among the groups (*χ*^2^ = 37.16, *p* < 0.0001), with CD patients scoring higher than HCs but lower than MDD patients. Correlation shows a positive association between GSRS and BDI-II scores in CD patients. Group-level fMRI analysis of normalized fALFF maps reveals significant clusters in the precuneus cortex. Subsequent seed-based connectivity analysis using the precuneus as seed region shows increased connectivity with the left supramarginal gyrus and decreased connectivity with the precuneus and anterior cingulate gyrus in MDD and CD compared to HCs.

**Conclusions:**

These exploratory findings reveal similar abnormalities in brain activity and connectivity in CD and MDD. A deeper understanding of these interactions may enable integrated treatment approaches that address both psychological and physiological aspects of these interconnected conditions.

## Plain language summary

Psychological stress is increasingly recognized as a key factor in Crohn’s disease (CD), and major depressive disorder (MDD) is a common comorbidity. Both conditions are linked to altered brain activity, suggesting shared biological mechanisms. However, these connections between the brain and gut remain poorly understood.

In this study, we used an advanced imaging scanner to examine brain activity and connectivity in individuals with CD, MDD, and healthy controls. Our findings reveal overlapping changes in brain regions in both CD and MDD patients, especially in areas related to self-awareness and emotion regulation.

These results provide early evidence of similar brain alterations in CD and MDD, supporting the idea that the gut and brain are closely linked. Understanding these connections may guide more effective and integrated treatment strategies that address both mental and physical health aspects of these conditions.

## Abstract (web summary)

Hartmann et al. use ultra-high field 7 T fMRI to investigate shared neurobiological mechanisms between Crohn’s disease (CD) and major depressive disorder (MDD). Their findings reveal overlapping abnormalities in brain activity and connectivity, suggesting a neuropsychological link that may inform integrated treatment strategies.

## Introduction

The central nervous system has a profound impact on complex processes, such as metabolism and immunity, at distant body sites^1^. This effect is particularly pronounced in inflammatory bowel disease (IBD), with several epidemiologic studies showing that stressful life events can trigger IBD flares^2–4^. Crohn’s disease (CD), one of the two major subtypes of IBD, represents a chronic inflammatory condition of the gastrointestinal tract, impacting millions worldwide, with a notable prevalence in developed nations^5^. Patients with CD exhibit higher incidences of MDD compared to the general population, and stressful life events have been linked to IBD flares, suggesting a bidirectional link^6^. However, the neurobiological correlates of stress-induced IBD exacerbations remain incompletely understood.

Neuroimaging technologies—particularly magnetic resonance imaging (MRI) and functional MRI (fMRI)—enable the precise investigation of altered brain structure and function underlying the (comorbid) psychiatric disorder. Blood oxygenation level dependent (BOLD) fMRI is a neuroimaging method that measures brain activity by indirectly detecting the associated changes in blood flow and blood oxygenation that follow neuronal activity^7^. Resting-state fMRI is an fMRI technique that measures the low-frequency fluctuations in the BOLD signal while the subject is in the resting condition (not actively performing any task)^8^. Compared to fMRI conducted at standard MRI field strengths, ultra-high field (UHF) 7 T fMRI offers significantly improved resolution with enhanced signal-to-noise (SNR) and contrast-to-noise (CNR) ratios, enabling more precise imaging^9–11^. This allows for the detection of neurostructural changes, connectivity alterations in smaller brain regions, and the identification of potential biomarkers that are not visible with lower magnetic field strengths. To the best of the authors’ knowledge, no research team has previously used 7 T fMRI to compare CD and MDD directly. Hence, the aim of this exploratory study is to precisely identify brain regions associated with CD and MDD, thereby paving the way for hypothesis generation to further understand these complex diseases, offering new perspectives on the bidirectional relationship.

Prior research has demonstrated associations between CD and MDD^2–4^. Moreover, various cerebral effects of CD have already been documented^12^. An fMRI study found that the midcingulate cortex (MCC) integrates afferent sensory information from the gut, as stress-evoked hyperactivity could be seen in the MCC in CD patients compared to controls^13^. Moreover, studies have found significant changes in grey matter structures and cortical thickness in CD patients. Bao et al. suggest that this can be partially explained by higher levels of depression and anxiety in these patients, as the brain regions involved influence pain, emotion, and homeostasis^14^. However, to date, very few fMRI studies have directly investigated the comorbidity of CD and MDD. For instance, Sun et al. compared CD patients with comorbid MDD or anxiety disorders to patients without these comorbidities and identified altered functional connectivity in amygdala subregions^15^. Such findings underscore the need for further research into the neural mechanisms linking these conditions. The present investigation examines whether functional brain changes are evident in both CD and MDD cohorts, potentially reflecting psychological comorbidities and shared neurobiological disruptions compared to healthy individuals. To explore this, resting-state functional magnetic resonance imaging (rs-fMRI) experiments were conducted using an ultra-high field 7 Tesla (7 T) MRI scanner in both patient groups alongside age and gender-matched HCs. The use of a 7 T scanner significantly improves the outcome of fMRI signals compared to those obtained at lower field strengths (resolution, SNR, and CNR), bringing considerable benefits to the study^10,11^. The objective centers on identifying shared neurobiological modifications in cerebral function between CD and MDD groups. Given the exploratory nature of the study and the lack of robust prior literature identifying specific brain regions jointly affected in CD and MDD, a purely data-driven approach was adopted. In this framework, fractional amplitude of low-frequency fluctuations (fALFF) was chosen as a first-pass modality to capture the intrinsic low-frequency oscillations in cerebral activity during the resting state. fALFF serves as a widely used neurophysiological index of spontaneous brain activity in resting-state fMRI data and has demonstrated improved specificity over traditional ALFF by reducing nonspecific physiological noise^16,17^. Our intent was not to assume fALFF as an optimal a priori modality, but to use it to empirically detect candidate regions of interest without predefining their spatial location. In the first step, regions exhibiting commonly altered BOLD signals in both the CD and MDD groups compared to HCs were identified using fALFF maps. In the subsequent step, a data-driven approach was applied using all of the significant clusters identified in the fALFF maps as seed regions for connectivity analysis. This approach enabled the identification of specific regions showing altered connectivity between different contrasts in the three observed groups (CD, MDD, and HC). These regions represent promising targets for advancing our understanding of how CD and MDD affect brain function and may provide insights into the neurobiological basis of their comorbidity. This sequential pipeline—fALFF followed by seed-based connectivity (SBC)—has been adopted in prior studies examining brain disorders in exploratory settings and serves here to identify overlapping neurofunctional disruptions without circular inference^18,19^.

Group comparisons of Beck Depression Inventory-II (BDI-II) scores show significantly higher levels of depressive symptoms in both MDD and CD patients compared to healthy controls. Resting-state fMRI analysis identified significantly reduced fALFF in the precuneus across both clinical groups. Follow-up SBC analysis using the precuneus as the seed region demonstrated convergent alterations in CD and MDD, characterized by an increase in connectivity with the left supramarginal gyrus and a decrease in connectivity with both the precuneus and the anterior cingulate cortex relative to controls.

## Methods

### Experimental model and study participant details

CD patients (*n* = 18, age = 29.79 ± 6.84; 14 males) along with age matched MDD patients (*n* = 18, age = 28.97 ± 5.96; 14 males), and HCs (*n* = 18, age = 28.35±, 5.23; 14 males) were included in the study (Table 1). The CD patients were recruited from the Clinic for Gastroenterology, Metabolic Diseases, and Internal Intensive Care Medicine (Medical Clinic III), Uniklinik Rheinisch-Westfälische Tehnische Hochschule (RWTH) Aachen, Germany. The CD patients were diagnosed based on colonoscopy and histology results, which were evaluated by experienced gastroenterologists in the clinic. In addition to the clinical diagnosis, gastrointestinal symptoms in CD patients were assessed via the Gastrointestinal Symptom Rating Scale (GSRS)^20^, licensed from AstraZeneca AB, Sweden. The GSRS questionnaire consists of 15 items, divided into five sub-scores. These sub-scores describe the symptom clusters of reflux, abdominal pain, indigestion, diarrhea, and constipation. A 7-point scale reflects the current status of the symptom severity profile, where 1 describes the absence of the symptom, and 7 is the highest symptom severity^21,22^. Moreover, the German version 6.0.0 of the Mini International Neuropsychiatric Interview (MINI) was performed to confirm the absence of MDD or other psychiatric disorders confounding the CD group^23^.

**Table 1:** Demographics table

Characteristics	CD (*n* = 18)	MDD (*n* = 18)	HC (*n* = 18)
Demographics	*n*%	*n*%	*n*%
Sex			
Female	4 22%	4 22%	4 22%
Male	14 78%	14 78%	14 78%
Age (years)	29.43 ± 7.44	29.04 ± 6.10	28.77 ± 6.65

Demographic data are presented for individuals with Crohn’s disease (CD), major depressive disorder (MDD), and healthy controls (HC).

The MDD patients were recruited from the Clinic for Psychiatry, Psychotherapy and Psychosomatics, Uniklinik RWTH Aachen. The MDD patients were diagnosed based on ICD-10 and DSM-5 criteria. The CD and HC groups were recruited based on having no history of neurologic or psychiatric disorders, as determined by MINI^23^. The handedness of all the subjects was assessed using the Edinburgh Handedness Inventory. Only right-handed subjects with no contraindications for 7 T MRI were included in the study. Additionally, depression symptom severity in all subjects was assessed using the German version of the BDI-II^24^. The BDI-II consists of 21 multiple-choice items, with four possible responses each.

### Statistical analysis

All statistical analysis was performed using the MATLAB (R2022a) software package. Differences in BDI-II scores were compared using a rank-based nonparametric Kruskal-Wallis test (*kruskalwallis* function in MATLAB). Subsequent post-hoc analyses were conducted using the *multcompare* function in MATLAB to determine the specific group differences. Within each Kruskal-Wallis test, the corrections for multiple comparisons were performed using the Bonferroni correction method with a significance level of 5%.

To explore associations between gastrointestinal symptoms and depression symptom severity in CD patients, a correlation analysis was performed between the GSRS total as well as the sub-scores and BDI-II scores, respectively. Spearman’s correlation coefficients were computed with a significance level of 5%. In all of the correlation analyses, the family-wise error rate (FWER), due to multiple comparisons, was controlled for using a permutation test^25^. A total of 1000 permutations were performed for each comparison (correlation), and the *p* value was adjusted using the “max statistics” method^25^.

### MRI data acquisition

MRI data acquisition was performed at Forschungszentrum Jülich using a 7 T Magnetom Terra scanner (Siemens Healthineers, Erlangen, Germany) equipped with a 1Tx/32Rx Head Coil 7 T Clinical (Nova Medical, Wilmington, MA, USA). Resting-state fMRI data were acquired using a 2D T2* weighted multiband accelerated echo planar imaging (EPI) sequence developed at the Center for Magnetic Resonance Research (CMRR), Minneapolis, MN, USA (https://www.cmrr.umn.edu/multiband/)^26–28^. The entire brain was covered with a field of view (FOV) of 220 × 220 mm^2^, a matrix size of 168 × 168, and a slice thickness of 1.3 mm. In total, 305 volumes with 100 slices each were acquired with a repetition time (TR) of 2000 ms, an echo time (TE) of 25 ms, and a flip angle (FA) of 70° using a multiband factor of 4. Subjects were instructed to close their eyes and not to fall asleep during the resting-state measurement. In addition, the lights in the scanner room were switched off during the entire resting-state measurement. To correct for susceptibility-induced geometric distortions, two additional fMRI volumes were recorded with opposite phase encoding direction (posterior-anterior phase-encoding).

Structural images were obtained using a T1-weighted MP2RAGE. The MP2RAGE acquires two gradient echo images with different inversion times (TI) and flip angles (FA) (inversion image 1 (INV1) TI = 840 ms, flip, FA = 4°, INV2 TI = 2370 ms, FA = 5°). The other sequence-related parameters were similar for both gradient echo images: echo time (TE) = 1.99 ms; repetition time (TR) = 4500 ms for signal-to-noise ratio (SNR) optimization. The image matrix was set to 320 × 300, achieving an isotropic resolution of 0.75 mm^3^ in 208 sagittal slices. The T1-weighted anatomical images referred to here were produced by combining the two gradient echo images by means of a ratio^29^.

### fMRI data preprocessing

The raw DICOM scans (structural and functional) were 3D converted into the Neuroimaging Informatics Technology Initiative (NIfTI) format using the dcm2niix tool^30^. The 3D structural and functional images were visually audited to check for poor scan quality, artifacts, and abnormal tissues using FSL View software (https://fsl.fmrib.ox.ac.uk/fsl/fslwiki/FslView). Due to poor quality fMRI scans and artifacts, data from five CD patients were excluded. This resulted in 13 CD patients (age = 29.43 ± 7.44; 9 males). To maintain balanced group sizes, reduce variance, and improve the reliability of statistical analyses, we correspondingly selected 13 age- and gender-matched participants from the MDD (age = 29.04 ± 6.10; 9 males) and HC (age = 28.77 ± 6.65; 9 males) groups. This matching aimed to control for potential confounding variables and enhance the interpretability of group comparisons.

The susceptibility-induced off-resonance field (fieldmap) was initially estimated using a method similar to that described in ref. ^31^ as implemented in FSL^32^. In the next step, the fMRI images were preprocessed using CONN^33^ release 22.a^34^, SPM^35^ release 12.7771 and MATLAB (R2022a).

Functional and anatomical data were preprocessed using a modular preprocessing pipeline^36^, which included the creation of voxel-displacement maps, realignment with susceptibility distortion correction using fieldmaps, slice timing correction, outlier detection, direct segmentation and MNI-space normalization, and smoothing. Functional data were realigned using the SPM realign and unwarp procedure^37^ with integrated fieldmaps for susceptibility distortion correction. All scans were co-registered to a reference image (first fMRI volume) using a least-squares approach and a 6-parameter (rigid body) transformation and were then resampled using b-spline interpolation^38^ to correct for motion, magnetic susceptibility geometric distortions, and their interaction simultaneously. Temporal misalignment between slices of the functional data was corrected using the SPM slice-timing correction (STC) procedure^39,40^ with sinc temporal interpolation to resample each slice’s BOLD timeseries to a common mid-acquisition time. Potential outliers were identified using ART^41^ based on framewise displacement above 0.9 mm or global BOLD signal changes above five standard deviations^42,43^. Outlier detection resulted in the removal of 0 to 26 fMRI volumes per subject (median = 0), with an average retention rate of 99% of volumes per subject (range = 91.48% to 100%). A reference BOLD image was computed for each subject by averaging all scans, excluding outliers. Both functional and anatomical data were normalized to standard MNI space, segmented into grey matter, white matter, and CSF tissue classes, and resampled to 1 mm isotropic voxels following a direct normalization procedure^43,44^ using the SPM unified segmentation and normalization algorithm^45,46^ with the default IXI-549 tissue probability map template. Direct normalization applies this procedure separately to the functional data—using the mean BOLD signal as the reference image—and to the structural data, using the raw T1-weighted volume as the reference image. Finally, functional data were smoothed using spatial convolution with a 4 mm full-width half-maximum (FWHM) Gaussian kernel.

Functional data were also denoised using a standard denoising pipeline^36^, which included the regression of potential confounding effects such as white matter timeseries (5 CompCor noise components), CSF timeseries (5 CompCor noise components), motion parameters and their first order derivatives (12 factors)^47^, outlier scans (below 26 factors)^42^, and linear trends (2 factors), within each functional run. This was followed by band-pass frequency filtering of the BOLD timeseries^48^ between 0.008 Hz and 0.09 Hz. CompCor^49,50^ noise components within white matter and CSF were estimated by computing the average BOLD signal as well as the largest principal components orthogonal to the BOLD average, motion parameters, and outlier scans within each subject’s eroded segmentation masks. Outlier detection resulted in the removal of 0 to 26 fMRI volumes per subject (median = 0), with an average retention rate of 99% of volumes per subject (range = 91.48% to 100%)^43^.

### fALFF - first and group-level analysis

Following preprocessing, rs-fMRI-fALFF was calculated using the processing pipelines from the CONN toolbox^33^. fALFF maps characterizing low-frequency BOLD signal variability at each voxel were estimated as the ratio between the root mean square (RMS) of the BOLD signal after denoising and band-pass filtering between 0.008 Hz and 0.09 Hz, divided by the same measure computed before band-pass filtering^17^. Finally, fALFF values across voxels were rank sorted and normalized separately for each individual subject using a Gaussian inverse cumulative distribution function with zero mean and unit variance.

To explore potential shared neurobiological changes in CD and MDD patients compared to HCs, a group-level general linear model (GLM)^36^-based analysis was performed on normalized fALFF maps. For each individual voxel, a separate GLM was estimated, with the fALFF value at this voxel as the dependent variable, and group BDI-II scores, age, and gender were set as the independent variables. The group analysis was performed to test the common differences between CD and MDD patients and HCs. The contrast was designed as [1 −0.5 −0.5 0 0 0] for HCs, CD patients, MDD patients, age, gender, and BDI-II score, respectively, to directly compare the average effect of CD and MDD patients against the HCs. Voxel-level hypotheses were evaluated using multivariate parametric statistics with random effects across subjects. Inferences were performed at the level of individual clusters (groups of contiguous voxels). Cluster-level inferences were based on parametric statistics from Gaussian Random Field theory^36,51^. Results were thresholded using a combination of a cluster-forming *p* < 0.01 voxel-level threshold and a familywise corrected p-FDR < 0.05 cluster-size threshold^52^. The rationale for utilizing a lower threshold (*p* < 0.01) at the voxel level was to enhance the detection of potential signals of interest, given the low sample size, while also acknowledging the potential for increased false positives. However, the subsequent application of a stringent correction for multiple comparisons at the cluster level (p-FDR < 0.05) ensured that only those clusters with a high degree of statistical reliability were considered significant. This two-level thresholding method facilitates the discovery of true differences in neural activity patterns while minimizing the risk of Type I errors. Significant clusters identified in the fALFF analysis were used as seed regions for subsequent seed-based functional connectivity analysis to investigate their region-level interactions.

### Seed-based connectivity - first and group-level analysis

To identify functional connectivity differences, whole-brain SBC maps were generated using seed regions defined by the entire significant cluster regions identified in the fALFF group comparison analysis. Functional connectivity strength was represented using Fisher-transformed bivariate correlation coefficients from a weighted GLM^36^. Functional connectivity strength was defined separately for each pair of seed and target voxels, modeling the association between their BOLD signal time series. In order to compensate for possible transient magnetization effects at the beginning of each run, individual scans were weighted using a step function convolved with an SPM canonical hemodynamic response function and rectified.

Group-level analysis was performed on SBC maps using separate GLM models to test differences between the HC group and the MDD group (HC > MDD), the HC group and the CD group (HC > CD), the HC group compared to a combined average of the MDD and CD groups (HC > 0.5MDD + 0.5CD), and regions with altered connectivity between the MDD group and CD group (MDD > CD)^36^. Within each model, a separate GLM was estimated for each individual voxel, with first-level SBC measures at this voxel as the dependent variable and the group BDI-II scores, age, and gender as the independent variables. Results were thresholded using a combination of a cluster-forming *p* < 0.001 voxel-level threshold and a familywise corrected p-FDR < 0.05 cluster-size threshold^52^. Corrections for multiple comparisons across the four separate GLM models (HC > MDD, HC > CD, HC > 0.5MDD + 0.5CD, and MDD > CD) were not performed in this analysis. This decision was based on the rationale that each model addresses a distinct hypothesis and is part of an exploratory investigation. Consequently, the findings should be interpreted as being preliminary and hypothesis-generating rather than confirmatory. Additionally, we placed a strong emphasis on the biological relevance and effect sizes of the findings, rather than relying solely on *p*-values for interpretation.

### Statistics and reproducibility

This study is exploratory in nature, and all statistical analyses were conducted in MATLAB (R2022a), SPM12, and the CONN toolbox v23a. A general linear model (GLM) framework was used for group-level analyses of both fALFF and seed-based connectivity (SBC) data. Group, age, gender, and BDI-II scores were included as covariates.

Voxel-wise inference was performed using parametric statistics with cluster-level correction based on Gaussian Random Field theory. A two-step thresholding procedure was applied: voxel-level thresholds of *p* < 0.01 for fALFF and *p* < 0.001 for SBC, followed by cluster-level FDR correction at p < 0.05. No correction was applied across contrasts due to the exploratory nature of the study.

All fMRI analyses were conducted on three matched groups of *n* = 13 (CD, MDD, and healthy controls), with no technical or biological replicates. Each participant contributed one resting-state fMRI scan; replicates were defined as individual subjects. To enhance reproducibility, standardized preprocessing pipelines were applied to all data, and quality control steps (e.g., outlier detection, motion correction) were uniformly implemented. Unthresholded statistical maps and *P*-value histograms are provided in the supplementary materials to promote transparency and reproducibility.

### Ethics statement

All participants signed informed consent prior to the study, and the study was conducted in accordance with the Declaration of Helsinki and approved by the Ethics Committee of the Faculty of Medicine of the RWTH Aachen University.

### Reporting summary

Further information on research design is available in the Nature Portfolio Reporting Summary linked to this article.

## Results

Rs-fMRI experiments were conducted on CD patients, MDD patients, and age- and gender-matched HCs using a 7 T MRI scanner. The use of high-resolution imaging aimed to identify shared neurobiological modifications in cerebral function across these groups by using fALFF as a neurophysiological marker and seed-based connectivity analysis to assess functional connectivity changes.

**Fig. 1:**
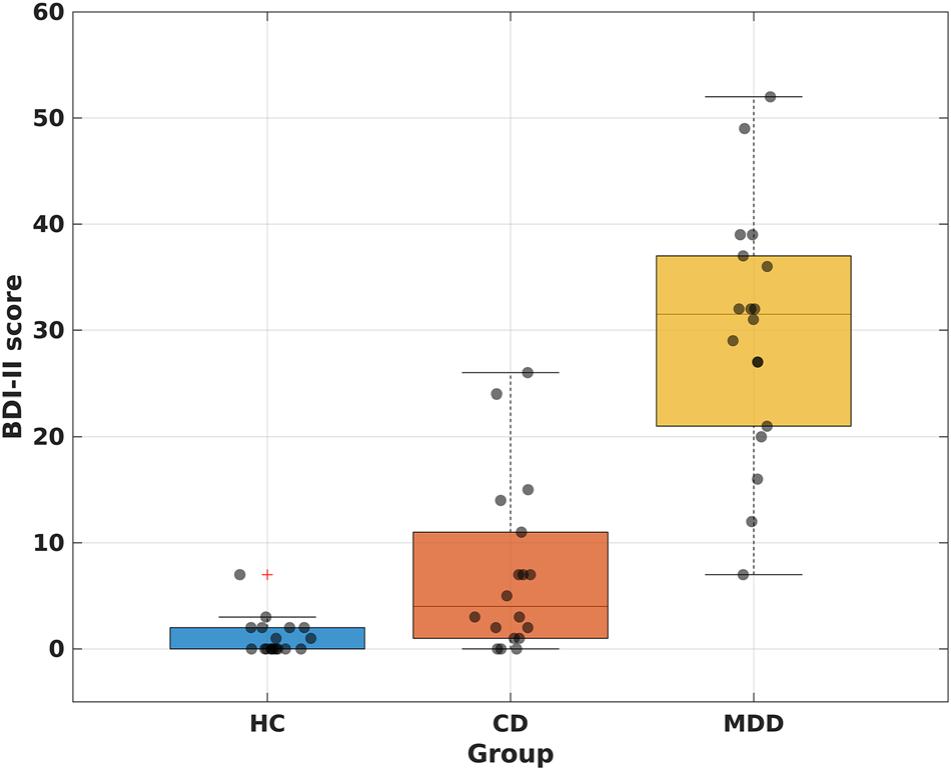
BDI-scores comparison of experimental groups. Box plot representation of the BDI-II scores from the three experimental groups. The Kruskal-Wallis test was performed to assess the differences in BDI-II scores among the groups (*n* = 18 in each group). A statistically significant difference was observed (*χ*² = 37.16, *p* < 0.0001), indicating that the BDI-II scores varied significantly across the groups. The central line within each box represents the median BDI-II score, while the box encompasses the interquartile range (IQR). Whiskers extend to the minimum and maximum values within 1.5 times the IQR.

Figure 1 shows the BDI-II scores from the three experimental groups. The Kruskal**–**Wallis test was performed to assess the differences in BDI-II scores among the groups. A statistically significant difference was observed (*χ*^2^ = 37.16, *p* < 0.0001).

### Association between the severity of digestive symptoms (GSRS) and depression (BDI-II) in CD patients

**Fig. 2:**
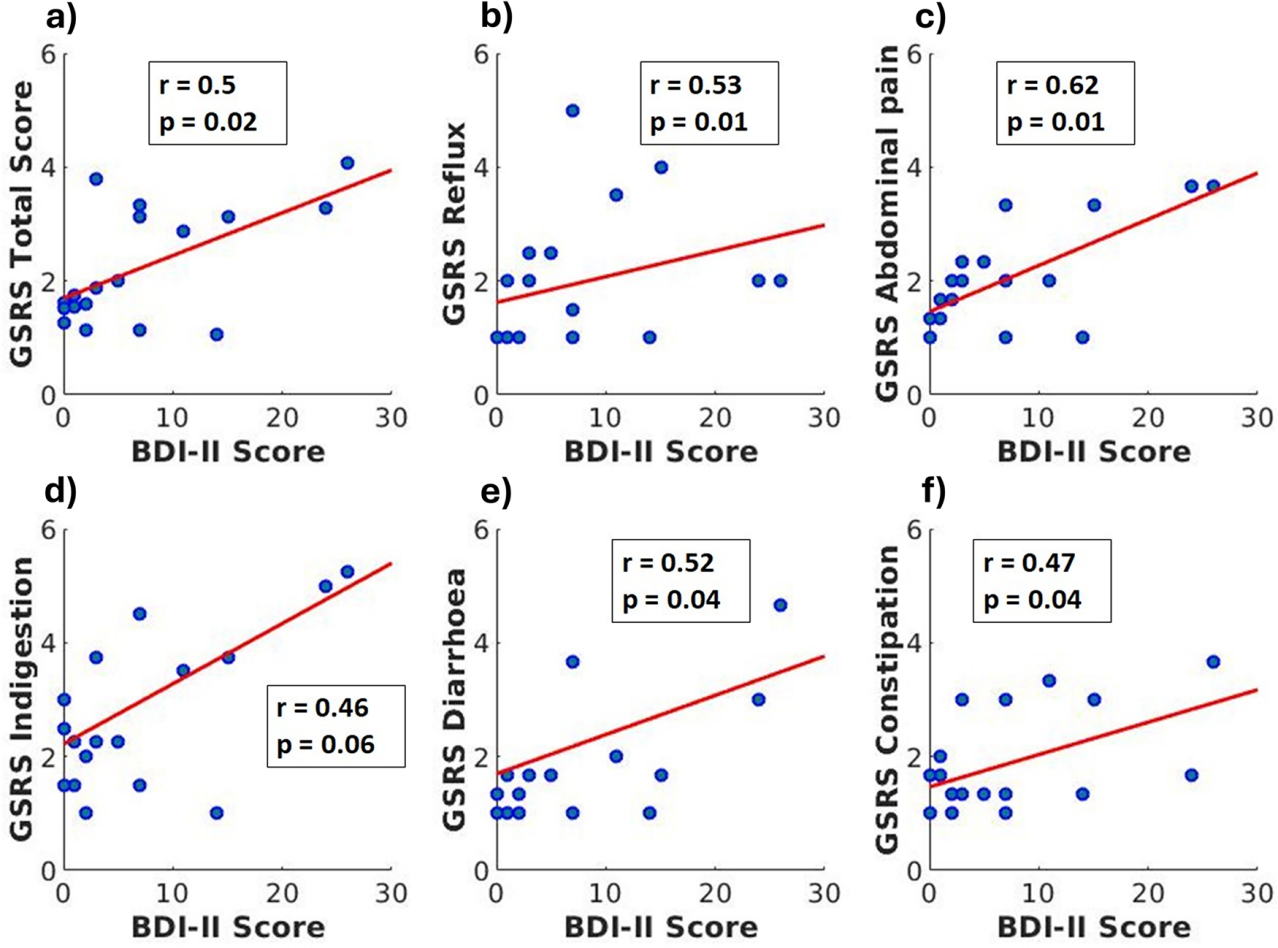
Correlation of GSRS and BDI-II scores. Scatter plots depicting the relationship between individual GSRS subscales and BDI-II scores (**a**–**f**). Each panel represents one specific GSRS domain: **a** total score, **b** reflux, **c** abdominal pain, **d** indigestion, **e** diarrhoea, **f** constipation. The association between GSRS and BDI-II score in CD patients (*n* = 17) was assessed via Spearman’s correlation analysis. Spearman’s correlation coefficients were computed with a significance level of 5%, and multiple comparisons were controlled for using a permutation test. Each blue color point represents the scores from an individual Crohn’s disease patient on both scales. The Spearman’s correlation coefficients of the observation (r) and *p* values, adjusted for multiple comparisons, are shown inside the boxes.

To explore associations between the GSRS and BDI-II in CD patients, a Spearman’s correlation analysis was performed between the total and sub-scores of the GSRS and the BDI-II scores. The Spearman’s correlation coefficients between the GSRS and BDI-II scores are shown in Fig. 2. The correlation analysis revealed a significant association between gastrointestinal symptoms and depression symptom severity in CD patients. A positive trend between high BDI-II scores and GSRS scores is evident in the scatter plot (Fig. 2).

### Results from neuroimaging

**Fig. 3:**
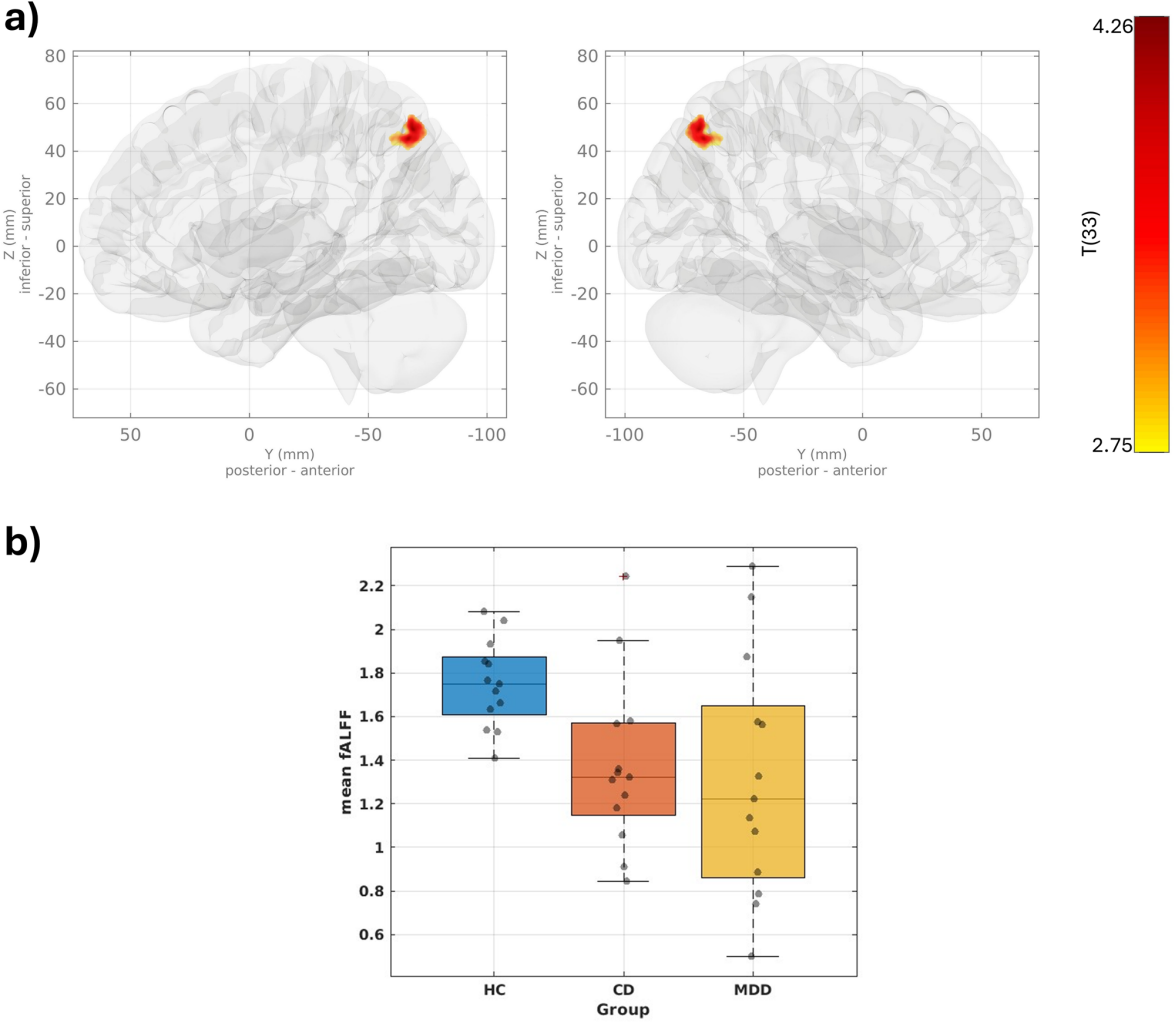
fALFF group-level analysis results. fALFF group-level analysis results showing a 3D glass-brain visualization of the cluster identified via GLM analysis (**a**). The boxplots illustrate the mean fALFF values inside the cluster for each group (**b**). A group-level GLM analysis was conducted on normalized fALFF maps. The contrasts were set as [1 −0.5 −0.5 0 0 0] for HCs, CD patients, MDD patients, age, gender, and BDI-II. Voxel-level hypotheses were evaluated using multivariate parametric statistics with random effects. Cluster-level inferences were based on Gaussian Random Field theory^36,51^. Clusters are shown in the left and right medial views of the glass brain. The boxplots depict the distribution of mean fALFF values within each group, with individual data points overlaid to show variability (*n* = 13 in each group).

The results of the GLM analysis on fALFF maps showed a significant cluster (446 voxels, p-unc 0.00028, p-FDR 0.030336) in the precuneus cortex region of the brain, with a peak cluster at the MNI coordinate (−02, −69, +49). The identified cluster is shown in Fig. 3. Both MDD and CD patients displayed decreased fALFF in the precuneus compared to HCs.

### Results from seed-based connectivity analysis

**Fig. 4:**
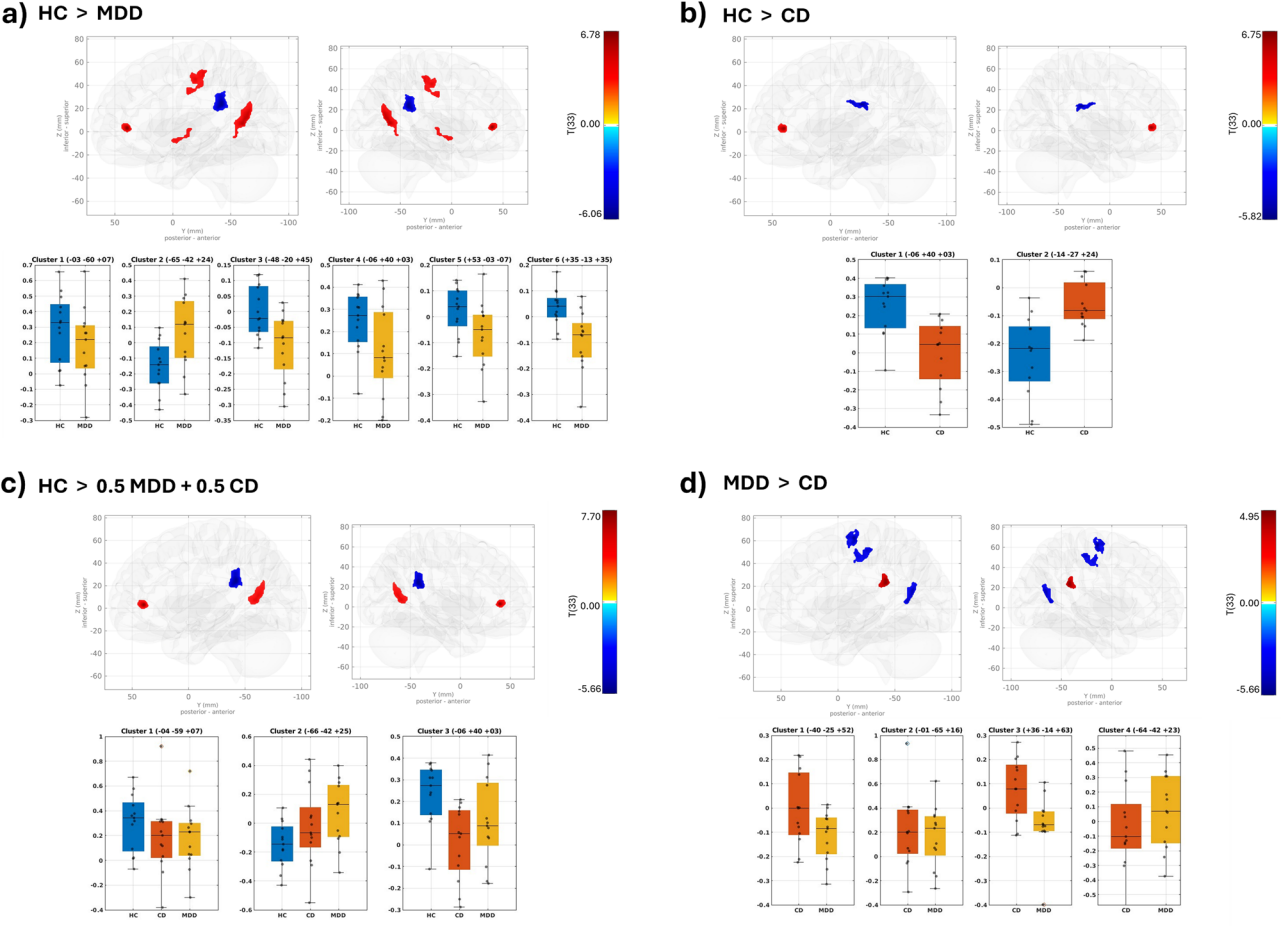
Group-level SBC analysis results. Group-level SBC analysis results showing a 3D glass-brain visualization of clusters identified via GLM analysis (top row within subplots) and boxplots displaying mean Fisher Z-transformed connectivity values within these clusters, stratified by group (*n* = 13 per group) (bottom row within subplots). Individual data points are overlaid on boxplots to illustrate within-group variability. Whole-brain SBC maps were generated using seed regions defined by the entire significant cluster regions identified in the fALFF group comparison analysis. Differences in connectivity are shown for **a** the HC group versus the MDD group (HC > MDD), **b** the HC group versus the CD group (HC > CD), **c** the HC group compared to a combined average of the MDD and CD groups (HC > 0.5MDD + 0.5CD), and **d** regions with altered connectivity between the MDD and CD groups (MDD > CD). Clusters are shown in the left and right medial views of the glass brain.

Group-level analysis was performed on these SBC maps using separate GLM models to test various contrasts: the HC group vs. the MDD group (HC > MDD), the HC group vs. the CD group (HC > CD), the HC group vs. the combined average of the MDD and CD groups (HC > 0.5MDD + 0.5CD), and regions with altered connectivity between the MDD and CD groups (MDD > CD). The results of these individual contrasts, showing the position of peak cluster, size, and *p* values are given in Supplementary Data 1. The identified cluster is shown in Fig. 4. These analyses showed regions with altered connectivity patterns in CD and MDD patients compared to HCs and are mainly evident in key areas such as the precuneus cortex, supramarginal gyrus, postcentral gyrus, anterior cingulate gyrus, and planum polare.

### HC > MDD contrast

Six significant clusters were identified in the HC > MDD contrast. The first cluster, with a peak at MNI coordinate (−03, −60, +07), primarily covered the precuneus cortex and showed decreased functional connectivity in MDD patients compared to HCs. The second cluster, peaking at (−65, −42, +24), was mainly located in the supramarginal gyrus (posterior division) and the parietal operculum cortex and exhibited increased connectivity in MDD patients compared to HCs. The third cluster, centered at (−48, −20, +45), and the sixth cluster, centered at (+35, −13, +35), predominantly covering the left and right postcentral gyri, showed decreased connectivity in MDD patients compared to HCs. The fourth cluster, located at (−06, +40, +03), covering the anterior cingulate gyrus (ACC), showed decreased connectivity in MDD. The fifth cluster, peaking at (+53, −03, −07), partly covering the planum polare, exhibited decreased connectivity in MDD patients.

### HC > CD contrast

Two significant clusters were identified. The first cluster was centered at (−06, +40, +03) and primarily covered the anterior cingulate gyrus. This cluster exhibited decreased connectivity between the precuneus and ACC in CD patients compared to HCs, mirroring the connectivity pattern observed in MDD patients. The second cluster peaked at MNI coordinates (−14, −27, +24), corresponding to an unlabeled region in the Harvard-Oxford atlas.

### HC > 0.5MDD + 0.5CD contrast

Three significant clusters emerged in the SBC group-level analysis using the precuneus as the seed region to identify common functional connectivity changes in the MDD and CD groups compared to the HC group. The first cluster, centered at (−04, −59, +07), showed decreased connectivity within the precuneus in MDD. The second cluster, centered at (−66, −42, +25), exhibited increased connectivity between the precuneus and the left supramarginal gyrus in MDD and CD patients compared to HCs. The third cluster, centered at (−06, +40, +03), showed decreased connectivity between the precuneus and the anterior cingulate gyrus in both MDD and CD patients compared to HCs.

### MDD > CD contrast

Four significant clusters were observed. The first cluster, centered at (−40, −25, +52), and the third cluster, centered at (+36, −14, +63), covered the postcentral gyrus left and precentral gyrus right. The second cluster, centered at (−01, −65, +16), predominantly covered the precuneus region. These three clusters showed increased connectivity in the CD group compared to the MDD group. The fourth cluster, peaking at (−64, −42, +23), was mainly located in the supramarginal gyrus (posterior division) and showed decreased connectivity in the CD group compared to the MDD group, similar to patterns observed in the contrast HC > MDD.

In studies with small sample sizes, the statistical power to detect true effects is inherently limited, increasing the risk of both false negatives and false positives. To address these challenges and enhance the interpretability of our findings, we have included unthresholded statistical maps and *p*-value histograms in Supplementary Figs. 1–5, as recommended in Sundermann et al.^53^.

## Discussion

This study aimed to uncover shared neurobiological alterations in CD and MDD patients by examining functional changes using rs-fMRI at an ultra-high field 7 T MRI scanner. The higher field strength offers decisive advantages, in particular an improved signal-to-noise ratio (SNR)^10^ and blood oxygen level-dependent (BOLD) contrast-to-noise ratio (CNR)^11,54^. The significantly improved resolution allows for more sensitive detection of potential changes, as has been shown in chronic inflammatory processes, but also in other fields, such as ischemia or dementia^55–58^. Such precise characterization of cerebral changes contributes to the novelty of this investigation. The bidirectional relationship between CD and MDD highlighted in our study aligns with previous research, emphasizing the interconnected nature of these diseases^59^. The effects of various antidepressants on the gut have been investigated in animal studies, demonstrating anti-inflammatory properties comparable to the effects of dexamethasone^60–63^. Clinically, similar observations have been made in humans, where IBD patients treated concurrently with antidepressants experienced lower relapse rates, reduced steroid usage, fewer endoscopies in the years following study enrolment, and even a selectively protective effect for CD^64,65^. On the other hand, advanced immune-targeted therapies, such as the anti-IL-12/23 antibody used in CD treatment, have also been associated with antidepressant effects that cannot be explained solely by improved physical health^66,67^. In addition, Koren et al. demonstrated that the activation of specific neurons in the insular cortex can even induce inflammation via memory traces^68^. A recent fMRI study identified altered brain activation patterns across different CD stages, suggesting that brain activity could possibly serve as a neurobiological biomarker for the disease^69^. Sun et al. investigated functional connectivity differences in CD patients with comorbid depression or anxiety compared to CD patients without psychiatric diagnoses and identified alterations in amygdala subregions^15^. While such studies highlight the impact of psychiatric comorbidity on brain function in CD, they do not disentangle whether the observed connectivity changes are driven by CD, the psychiatric condition, or their interaction. In contrast, our study examined CD and MDD groups independently, excluding individuals with comorbid diagnoses. This approach allowed for a more targeted investigation of disease-specific neural alterations. To the best of our knowledge, this is one of the first studies to concurrently examine both CD and MDD as separate clinical groups within the same neuroimaging framework to explore potential shared neurobiological mechanisms. Given the high prevalence of anxiety disorders as an additional comorbidity in CD^59^, future studies should extend this approach to include anxiety in order to develop a more comprehensive model of neuropsychological involvement in inflammatory bowel disease^12,59^. We investigated whether functional changes, specifically variations in BOLD signals and functional connectivity, were manifest in both CD and MDD cohorts compared to healthy individuals. To investigate this, rs-fMRI experiments were conducted using ultra-high field 7 T MRI scanners with fALFF as a neurophysiological marker. Our analysis included group-level GLM to assess fALFF maps and seed-based connectivity (SBC) maps. The results revealed significant clusters with altered common connectivity patterns in both CD and MDD patients, highlighting potential shared neurobiological mechanisms underlying these conditions. The findings from the various contrasts among the three groups are discussed below.

Post-hoc analyses showed significant differences in the BDI-II score between HCs and MDD patients (*p* = 0.0041) and between CD patients and MDD patients (*p* < 0.001). Although the CD patients were not diagnosed with MDD, our results show that they are ranked between “minimal or mild depression” in the standardized cut-offs for the BDI-II.

To aid further understanding of the relationship between MDD and CD, correlation analysis between GSRS and BDI-II was performed. Both test scores provide an estimate of the symptom severity of the disease. Our results show a positive trend between high BDI-II scores and the symptom severity in each sub-score of GSRS (reflux, abdominal pain, indigestion, diarrhea, and constipation). These findings are consistent with previous studies demonstrating a strong comorbidity between IBD and MDD^70,71^. These correlations indicate that our rather small cohort demonstrates the widely observed comorbidity, and first implications can be derived from further statistical analysis to generate hypotheses for further research in this direction.

Evidence from neuroimaging showed shared neurobiological changes in MDD and CD. The group-level GLM analysis performed on fALFF maps revealed significant cluster differences between the three groups. The results show that MDD patients and CD patients share decreased fALFF in the precuneus compared to HCs. The precuneus is located in the parietal lobe and is an important component of the default mode network (DMN)^72^. The DMN describes a network with high functional connectivity during rest and is linked to non-goal-directed processes^73,74^. The DMN is also involved in the consolidation of memory, working memory^74^, the continuous sampling of external and internal environments^72^, cognitive and emotional processing and functioning^75^, and self-referential mental processes^76–79^.

Our observation of decreased fALFF in precuneus in MDD patients replicates previous studies showing altered activity in the PCC/precuneus region in patients exhibiting depressive symptoms or diagnosed with MDD^80–85^. Moreover, significant findings highlight changes in the precuneus region, notably its associations with Alzheimer’s disease^86,87^ and attention deficit hyperactivity disorder (ADHD)^73,88^. These conditions are likewise strongly linked to an increased risk of depressive symptoms^89–91^. Task-based fMRI studies also emphasize the role of the precuneus in MDD, as performing self-referential tasks leads to altered activation and connectivity in the precuneus region in MDD patients^92^. In terms of CD patients, recent functional MRI studies have demonstrated that ALFF values from the precuneus can serve as a neurobiomarker for predicting the current disease activity^69,93^. By conducting a direct comparison between patients with MDD and CD, our results emphasize the potentially critical role of the precuneus in the comorbidity of these conditions.

The SBC analysis revealed shared and unique functional links with the precuneus. The six significant clusters identified in the HC > MDD contrast are the precuneus, supramarginal gyrus/parietal operculum, postcentral gyri, planum polare, and ACC. The precuneus showed decreased functional connectivity in MDD patients compared to HCs. The supramarginal gyrus (posterior division) and the parietal operculum cortex exhibited increased connectivity in MDD patients compared to HCs. The precentral and postcentral gyri, planum polare, and ACC showed decreased connectivity in MDD patients compared to HCs.

Previous research comparing MDD and HC has identified a similar pattern of altered connectivity, particularly in the precuneus, which has been associated with symptom severity in MDD. This hypoconnectivity in MDD patients may indicate a failure in processing negative memories and emotions^83,94–96^. Additionally, earlier studies have shown an increased amplitude of low-frequency fluctuation (ALFF) and functional connectivity in the supramarginal gyrus and parietal operculum among individuals with MDD^97^. Regions such as the postcentral gyrus, supramarginal gyrus, and cingulate gyrus have also been implicated in the onset of MDD symptoms^98^. Notably, the ACC also showed significantly decreased connectivity in the HC > CD contrast, warranting further discussion in the following paragraph.

Subsequently, we examined the HC > CD contrast in the SBC analysis. The significant cluster in the ACC showed reduced connectivity with the precuneus in CD patients compared to healthy controls, mirroring the connectivity pattern also observed in MDD patients.

The cingulate gyrus is considered part of the limbic lobe and is, therefore, a part of sensory, action, and attentional control, decision making^99^, and emotional regulation in depression^100^. The anterior part of cingulate gyrus has been identified as a consistently altered region within the limbic lobe in several studies, with changes associated with both MDD and treatment responses^101,102^. Similarly, Rubart et al. observed decreased functional connectivity between the precuneus and cingulate gyrus, which was associated with symptom severity in MDD patients^83^. Additionally, in task-based fMRI studies, the ACC also presents as being important to MDD-linked regions^103^. The comparison of resting-state and task-based fMRI in MDD patients showed an increased negative correlation in the precuneus and cingulate gyrus, suggesting dysfunctional network dynamics within the DMN^104^. This finding highlights the importance of resting-state studies for a deeper understanding of brain networks.

The ACC has been recognized as a neurobiological correlate of both CD and MDD, with connectivity alterations that may differentiate irritable bowel syndrome (IBS) patients with and without MDD^105^. Moreover, structural, functional, and metabolic changes in the anterior cingulate gyrus in CD patients were found to be linked with the onset of MDD and CD symptoms^106^. As our findings replicate the previous literature while comparing both diseases in a one-study approach, they add weight to the suggestion that the ACC might be involved in the neurobiological mechanisms underlying depression-related comorbidity in CD patients. The observed hypoconnectivity between the precuneus and ACC may reflect deficits in emotional regulation in MDD, with a smaller effect size in CD, which corresponds to the milder depressive symptoms typically seen in CD patients.

The HC > 0.5MDD + 0.5CD contrast revealed shared functional connectivity changes across CD and MDD in the SBC analysis. Using the precuneus as the seed region revealed three significant clusters in MDD and CD patients compared to HCs. Increased connectivity was found between the precuneus and the left supramarginal gyrus, while decreased connectivity was observed within the precuneus and between the precuneus and the ACC.

These results are particularly interesting, as the patterns of beta values (connectivity strength) shown in Fig. 4C reveal a trend whereby the connectivity strength in the CD group lies between that of the MDD and HC groups across all clusters. This observation suggests a similar direction of altered connectivity between the MDD and CD groups, indicating potential shared neurobiological disruptions. The regions identified in this analysis also emerged in the HC > MDD contrast, aligning with previous studies that highlight common connectivity changes in both conditions. These studies have investigated brain changes in MDD and CD independently, with the main effects in each condition being replicated many times, aligning with our findings. Notably, the precuneus region has consistently emerged in previous studies focusing on MDD^80–85^. Given that MDD is a frequently observed comorbidity in IBD, where the underlying pathophysiology also remains unclear, our research highlights critical brain regions that may help identify the key mechanisms underlying this association^65^.

Consistent with prior findings, these results suggest that both MDD and CD are characterized by increased functional connectivity in regions associated with emotional regulation, sensory processing, and attention. These shared neurobiological changes may help explain the increased vulnerability of CD patients with depressive symptoms to more frequent relapses and a greater need for medication compared to CD patients without depressive symptoms^65^.

The SBC analysis of the MDD > CD contrast identified four significant clusters with differing connectivity patterns between CD and MDD patients. Compared to the MDD group, the CD group exhibited reduced connectivity in the supramarginal gyrus (posterior division). Conversely, the other clusters, mainly covering the precuneus cortex, left postcentral gyrus, and right precentral gyrus, displayed increased connectivity in the CD group compared to the MDD group.

The cluster located in the posterior division of the supramarginal gyrus exhibited increased connectivity in the MDD group compared to the CD group, similar to patterns observed in the contrast HC > MDD. This increased connectivity in the supramarginal gyrus is possibly attributable to its critical role in overcoming emotional egocentricity in social judgment and self-referential processing, distinguishing individuals with MDD from those with CD and HCs^107^.

Other significant clusters that displayed increased connectivity in the CD group compared to the MDD group included the precuneus cortex, postcentral gyrus, and precentral gyrus. The postcentral gyrus functions as the primary somatosensory cortex, processing sensory information from the body, while the precuneus is linked to self-referential thinking and consciousness^83,94–96,108^. The increased connectivity in these regions in CD patients suggests that CD may involve distinct alterations in sensory processing and self-referential cognition, potentially differentiating it from the more emotion-focused disruptions observed in MDD patients. These interpretations are based on exploratory findings and require further investigation in future studies.

Several limitations of this study must be considered. First, its cross-sectional design precludes establishing causality between brain abnormalities and the origins of the conditions; we can only infer associations. Additionally, the relatively small sample size of 13 subjects per group limits the generalizability and robustness of our findings. Future studies with larger sample sizes are needed to enhance the reliability of the results, while longitudinal designs would better support the assessment of causal relationships. Importantly, our study is exploratory in nature and should be interpreted as hypothesis-generating. While we observed parallel reductions in precuneus fALFF and altered precuneus–ACC connectivity in both patient groups, the absence of significant imaging–clinical correlations or within-subject associations limits the conclusions that can be drawn regarding shared mechanisms. Future work should include longitudinal designs and larger cohorts, allowing for mediation analyses and stronger causal inference. Another relevant consideration is the frequent comorbidity of anxiety disorders in individuals with CD^59^. Although all participants were systematically screened using the MINI, and individuals with manifest psychiatric disorders, including anxiety disorders, were excluded, the possibility of subclinical anxiety symptoms in the CD group cannot be entirely ruled out. Future studies would benefit from explicitly assessing anxiety severity to further elucidate its potential contribution to alterations in brain connectivity in CD. Another consideration is the potential influence of medication on brain connectivity and structure, which was not addressed in this study, but should be considered in future research. Furthermore, distinguishing between active and inactive phases of CD in future studies could provide more nuanced insights into the brain’s role in the disease’s progression and symptomatology. Addressing these factors in future investigations will provide a more comprehensive understanding of the neurobiological underpinnings of CD and its comorbidity with MDD.

## Conclusion

This exploratory 7 T rs-fMRI study provides valuable first insights into shared neurobiological changes in CD and MDD patients compared to HC’s. The advantage of utilizing 7 T fMRI in this study stems from its key benefits, as previously mentioned, specifically, the higher field strength enhances resolution by providing greater SNR and CNR^10,11,54^. Our findings of aberrant brain regions in both CD and MDD patients present the first steps towards finding possible neurobiological correlates of the comorbidity, providing a basis for generating hypotheses for future research. Understanding these shared neural mechanisms is crucial for developing integrated therapeutic strategies that address both conditions simultaneously. If the neural substrates of CD and MDD observed here can be replicated in larger samples, this could highlight the potential benefit of incorporating psychological diagnostics and support into the treatment of IBD patients. However, given the sample size, further verification in larger studies is needed. Utilizing multimodal data, including multi-omics and clinical assessment data, future research should aim to verify these shared changes and explore their possible contributions to the pathophysiology of both disorders. This may help determine whether the shared neural alterations in CD and MDD patients originate from a common underlying mechanism or whether one condition contributes to the onset of the other. Additionally, further investigation into the effects of antidepressants on CD could provide valuable insights for developing more effective, holistic treatment approaches. Another important consideration is that anxiety disorders also show a high comorbidity with CD; therefore, the potential impact of subclinical anxiety on our results should not be overlooked. Consequently, including additional measures to distinguish MDD symptoms and specifically assess anxiety would contribute to a more comprehensive picture.

Future studies with larger sample sizes could help bridge the gap between animal model research and clinical applications in humans, ultimately contributing to a deeper understanding of the pathophysiology of both diseases and the potential mechanisms underlying the comorbidity.

## Supplementary information

Supplementary Information

DOASF

Supplementary Data 1

Supplementary Data 2

Reporting Summary

## Data Availability

The source data for Figs. 1, 2 and 3c can be found in Supplementary Data 2 respectively.
