# Supplementary material for: Shared neurobiological changes in individuals with Crohn’s disease and major depressive disorder
Source: Commun Med (Lond). 2025 Sep 17;5:388. doi: 10.1038/s43856-025-01117-w (PMC12443968; doi:10.1038/s43856-025-01117-w)
Supplement: Supplementary file 1 — Supplementary Information [file 43856_2025_1117_MOESM1_ESM.pdf]

Shared neurobiological changes in individuals with Crohn's disease and Major Depressive Disorder

Hanna A Hartmann[1,2], Marja L Berthold[1,2], Shukti Ramkiran[1,2,3], Lukas Bündgens[4], Julius W Jaeger[4], Jana Hagen[1,2], Maria Backhaus[4], Maria Collée[1], Gereon J Schnellbächer[1,2], Tanja Veselinović[1,2], N. Jon Shah[1,8,9,10], Kai M Schneider[5,6,7], Ravichandran Rajkumar[1,2,3,8*,#], Irene Neuner[1,2,3,8*,#]

[1]Department of Psychiatry, Psychotherapy and Psychosomatics, RWTH Aachen University, 52074 Aachen, Germany

[2]Institute of Neuroscience and Medicine 4, INM-4, Forschungszentrum Jülich, 52425 Jülich, Germany

[3]Center for Computational Life Science, RWTH Aachen University, 52074 Aachen, Germany

[4] Department of Gastroenterology, Metabolic Diseases and Internal Intensive Care Medicine, Uniklinik RWTH Aachen, Aachen, Germany

[5]Department of Medicine 1, University Hospital Carl Gustav Carus Dresden, Technische Universität (TU), Fetscherstrasse 74, 01307, Dresden, Germany

[6]Else Kroener Fresenius Center for Digital Health, Medical Faculty Carl Gustav Carus, TUD Dresden University of Technology, Dresden, Germany

[7]Center for Regenerative Therapies Dresden (CRTD), Technische Universität (TU), Dresden, Germany

[8] JARA-BRAIN, 52074 Aachen, Germany

[9]Department of Neurology, RWTH Aachen University, 52074 Aachen, Germany

[10]Institute of Neuroscience and Medicine 11, INM-11, Forschungszentrum Jülich, 52425 Jülich Germany

* These authors jointly supervised this work

# corresponding authors

Email: Ravichandran Rajkumar rrajkumar@ukaachen.de

Irene Neuner ineuner@ukaachen.de

Unthresholded Statistical Maps and P-Value Histograms

Unthresholded statistical maps provide a comprehensive visualization of the entire spectrum of statistical values across the brain, not just those surpassing a predefined significance threshold. This approach allows for the identification of subthreshold effects that may be consistent with known functional networks or anatomical structures, offering insights that might be overlooked when focusing solely on thresholded results.

Supplementary Figure 1: Unthresholded Statistical Maps and P-Value Histograms for fALFF analysis [HC > 0.5(MDD + CD)]

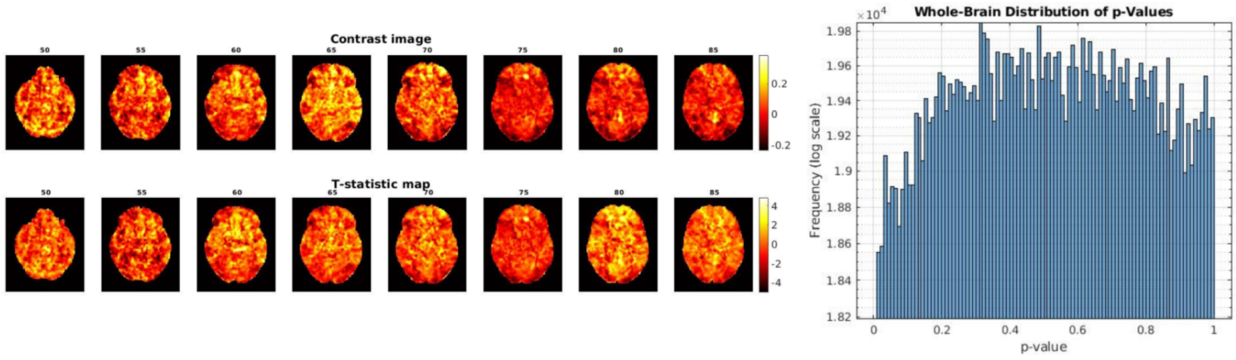

Supplementary Figure 1: Unthresholded Statistical Maps (left) and P-Value Histograms (right) for fALFF analysis with HC > 0.5(MDD + CD) contrast. All maps are displayed in axial slices across the whole brain.

Supplementary Figure 2: Unthresholded Statistical Maps and P-Value Histograms for SBC analysis [HC > MDD]

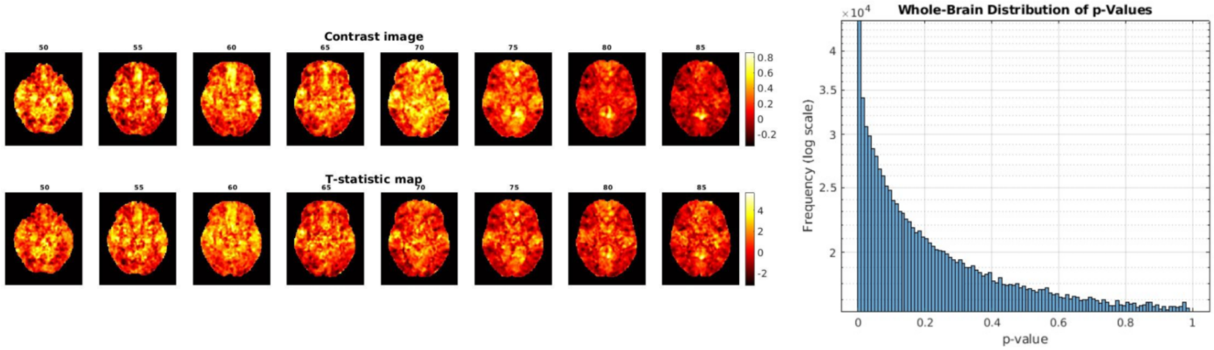

Supplementary Figure 2: Unthresholded Statistical Maps (left) and P-Value Histograms (right) for seed-based connectivity (SBC) analysis with the HC > MDD contrast. All maps are displayed in axial slices across the whole brain.

Supplementary Figure 3: Unthresholded Statistical Maps and P-Value Histograms for SBC analysis [HC > CD]

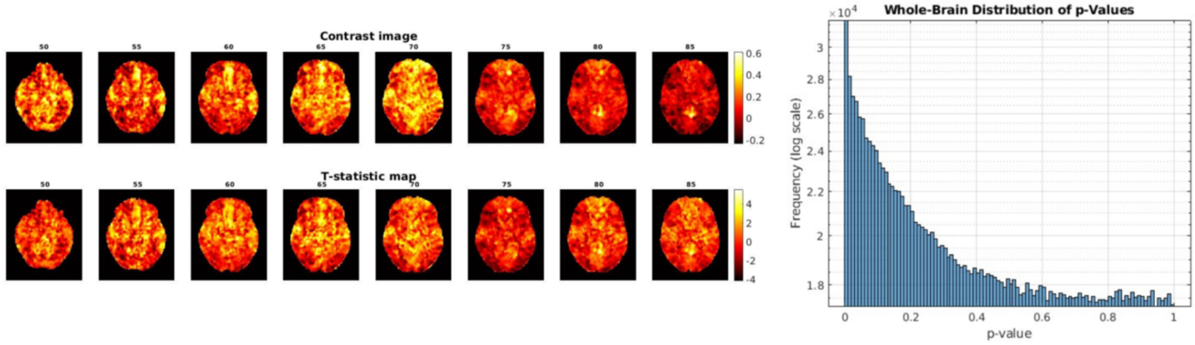

Supplementary Figure 3: Unthresholded Statistical Maps (left) and P-Value Histograms (right) for seed-based connectivity (SBC) analysis with the HC > CD contrast. All maps are displayed in axial slices across the whole brain.

Supplementary Figure 4: Unthresholded Statistical Maps and P-Value Histograms for SBC analysis [HC > 0.5(MDD + CD)]

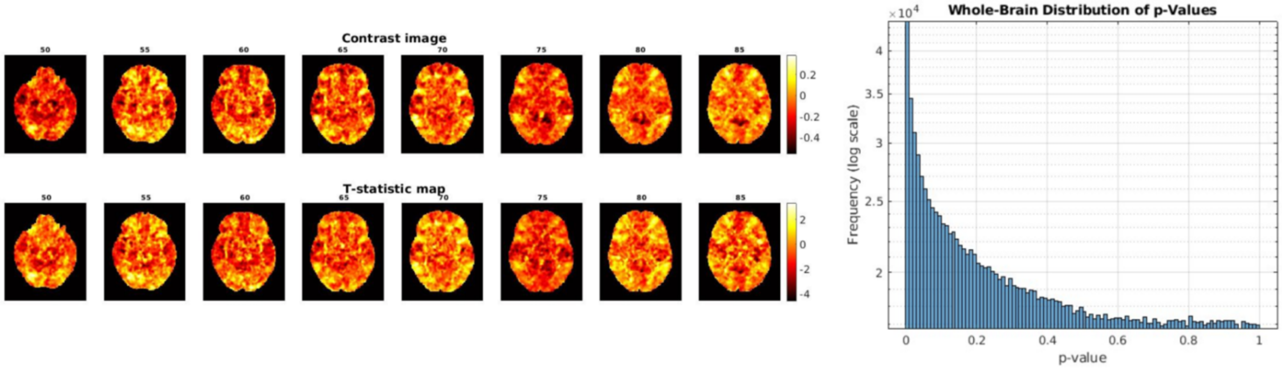

Supplementary Figure 4: Unthresholded Statistical Maps (left) and P-Value Histograms (right) for seed-based connectivity (SBC) analysis with the HC > 0.5(MDD + CD) contrast. All maps are displayed in axial slices across the whole brain.

Supplementary Figure 5: Unthresholded Statistical Maps and P-Value Histograms for SBC analysis [MDD > CD]

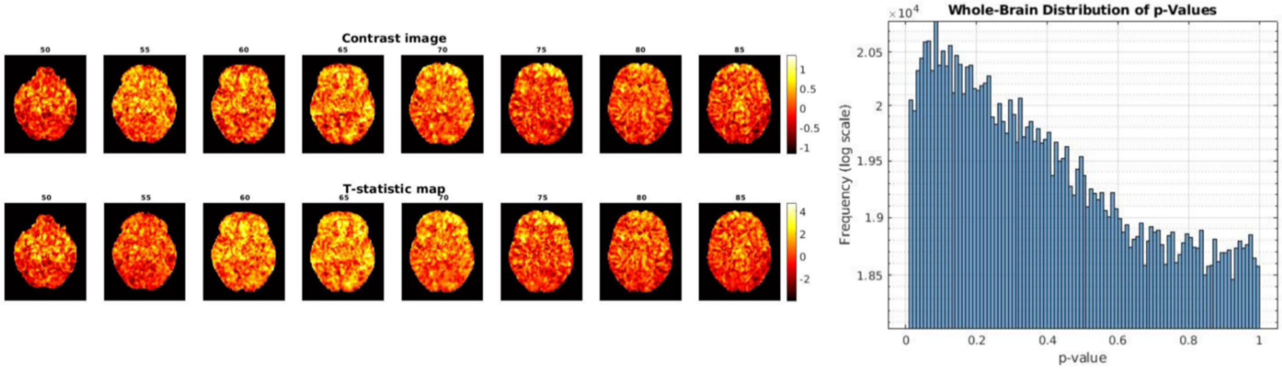

Supplementary Figure 5: Unthresholded Statistical Maps (left) and P-Value Histograms (right) for seed-based connectivity (SBC) analysis with the MDD > CD contrast. All maps are displayed in axial slices across the whole brain.
