# Supplementary material for: Shared neurobiological changes in individuals with Crohn’s disease and major depressive disorder
Source: Commun Med (Lond). 2025 Sep 17;5:388. doi: 10.1038/s43856-025-01117-w (PMC12443968; doi:10.1038/s43856-025-01117-w)
Supplement: Supplementary file 2 — DOASF [file 43856_2025_1117_MOESM2_ESM.pdf]

Description of Additional Supplementary Files

File name- Supplementary Data 1

File description – Group level analysis results of seed-based connectivity maps.

File name- Supplementary Data 2

File description – Source Data for Figures 1,2 and 3(c).
