# Supplementary material for: Shared neurobiological changes in individuals with Crohn’s disease and major depressive disorder
Source: Commun Med (Lond). 2025 Sep 17;5:388. doi: 10.1038/s43856-025-01117-w (PMC12443968; doi:10.1038/s43856-025-01117-w)
Supplement: Supplementary file 3 — Supplementary Data 1 [file 43856_2025_1117_MOESM3_ESM.pdf]

Supplementary Table 1: Group level analysis results of seed based connectivity maps

Cluster (x,y,z)	size	size p-FWE	size p-FDR	size p-unc	peak p-FWE	peak p-unc	Regions
**Contrast HC > MDD**							
(-03 -60 +07)	1145	0.000001	0.000002	0.000000	0.439646	0.000001	940 voxels (82%) covering 2% of Precuneous (Precuneous Cortex) with center at (-3,-62,+14)<br>54 voxels (5%) covering 0% of LG l (Lingual Gyrus Left) with center at (-3,-61,+6)<br>42 voxels (4%) covering 1% of Ver45 (Vermis 4 5) with center at (-2,-56,+4)<br>37 voxels (3%) covering 1% of Cereb45 l (Cerebelum 4 5 Left) with center at (-5,-54,-3)<br>22 voxels (2%) covering 0% of ICC l (Intracalcarine Cortex Left) with center at (-3,-65,+12)<br>2 voxels (0%) covering 0% of ICC r (Intracalcarine Cortex Right) with center at (+1,-64,+11)<br>2 voxels (0%) covering 0% of SCC l (Supracalcarine Cortex Left) with center at (-1,-67,+15)<br>46 voxels (4%) covering 0% of not-labeled with center at (+0,-61,+9)
(-65 -42 +24)	983	0.000007	0.000005	0.000000	0.327547	0.000001	394 voxels (40%) covering 5% of pSMG l (Supramarginal Gyrus, posterior division Left) with center at (-63,-42,+25)<br>167 voxels (17%) covering 4% of PO l (Parietal Operculum Cortex Left) with center at (-60,-38,+22)<br>162 voxels (16%) covering 2% of aSMG l (Supramarginal Gyrus, anterior division Left) with center at (-65,-38,+28)<br>23 voxels (2%) covering 1% of PT l (Planum Temporale Left) with center at (-61,-38,+19)<br>237 voxels (24%) covering 0% of not-labeled with center at (-67,-41,+25)
(-48 -20 +45)	624	0.000521	0.000268	0.000005	0.991528	0.000017	410 voxels (66%) covering 1% of PostCG l (Postcentral Gyrus Left) with center at (-44,-22,+47)<br>148 voxels (24%) covering 0% of PreCG l (Precentral Gyrus Left) with center at (-34,-23,+49)<br>66 voxels (11%) covering 0% of not-labeled with center at (-31,-24,+47)
(-06 +40 +03)	314	0.045462	0.017933	0.000437	0.066163	0	247 voxels (79%) covering 1% of AC (Cingulate Gyrus, anterior division) with center at (-7,+40,+4)<br>18 voxels (6%) covering 0% of PaCiG l (Paracingulate Gyrus Left) with center at (-9,+43,+3)<br>49 voxels (16%) covering 0% of not-labeled with center at (-11,+39,+4)

Supplementary Table 1: Group level analysis results of seed based connectivity maps

(+53 -03 -07)	248	0.133628	0.044228	0.001348	0.999999	0.000067	44 voxels (18%) covering 1% of PP r (Planum Polare Right) with center at (+52,-4,-6)<br>21 voxels (8%) covering 1% of aSTG r (Superior Temporal Gyrus, anterior division Right) with center at (+56,-1,-8)<br>2 voxels (1%) covering 0% of HG r (Heschl's Gyrus Right) with center at (+54,-13,+2)<br>181 voxels (73%) covering 0% of not-labeled with center at (+53,-10,-4)
(+35 -13 +35)	235	0.165632	0.046528	0.001702	0.999993	0.000054	133 voxels (57%) covering 1% of PostCG r (Postcentral Gyrus Right) with center at (+42,-23,+40)<br>9 voxels (4%) covering 0% of PreCG r (Precentral Gyrus Right) with center at (+38,-16,+36)<br>93 voxels (40%) covering 0% of not-labeled with center at (+36,-14,+35)
**Contrast HC > CD**							
(-06 +40 +03)	352	0.024913	0.022202	0.000237	0.070192	0	244 voxels (69%) covering 1% of AC (Cingulate Gyrus, anterior division) with center at (-7,+41,+3)<br>69 voxels (20%) covering 1% of PaCiG l (Paracingulate Gyrus Left) with center at (-8,+43,+2)<br>39 voxels (11%) covering 0% of not-labeled with center at (-11,+39,+3)
(-14 -27 +24)	320	0.041298	0.022202	0.000396	0.513196	0.000002	320 voxels (100%) covering 0% of not-labeled with center at (-12,-25,+24)
**Contrast HC > 0.5 MDD + 0.5 CD**							
**Cluster (x,y,z)**	**size**	**size p-FWE**	**size p-FDR**	**size p-unc**	**peak p-FWE**	**peak p-unc**	**Regions**
(-04 -59 +07)	853	0.000031	0.000029	0	0.621711	0.000002	734 voxels (86%) covering 2% of Precuneous (Precuneous Cortex) with center at (-3,-61,+13)<br>49 voxels (6%) covering 0% of LG l (Lingual Gyrus Left) with center at (-4,-61,+6)<br>18 voxels (2%) covering 0% of Ver45 (Vermis 4 5) with center at (-2,-56,+6)<br>14 voxels (2%) covering 0% of ICC l (Intracalcarine Cortex Left) with center at (-3,-64,+11)<br>1 voxels (0%) covering 0% of ICC r (Intracalcarine Cortex Right) with center at (+1,-64,+12)<br>37 voxels (4%) covering 0% of not-labeled with center at (+0,-61,+9)

Supplementary Table 1: Group level analysis results of seed based connectivity maps

(-66 -42 +25)	828	0.000041	0.0.000029	0	0.0.650932	0.0.000003	359 voxels (43%) covering 4% of pSMG l (Supramarginal Gyrus, posterior division Left) with center at (-64,-43,+25)<br>119 voxels (14%) covering 2% of aSMG l (Supramarginal Gyrus, anterior division Left) with center at (-65,-39,+27)<br>113 voxels (14%) covering 3% of PO l (Parietal Operculum Cortex Left) with center at (-61,-38,+22)<br>10 voxels (1%) covering 0% of PT l (Planum Temporale Left) with center at (-61,-38,+19)<br>227 voxels (27%) covering 0% of not-labeled with center at (-67,-41,+25)
(-06 +40 +03)	437	0.006888	0.003227	0.000065	0.007174	0	296 voxels (68%) covering 1% of AC (Cingulate Gyrus, anterior division) with center at (-7,+41,+4)<br>60 voxels (14%) covering 1% of PaCiG l (Paracingulate Gyrus Left) with center at (-9,+43,+3)<br>81 voxels (19%) covering 0% of not-labeled with center at (-11,+39,+4)

Contrast MDD > CD

Cluster (x,y,z)	size	size p-FWE	size p-FDR	size p-unc	peak p-FWE	peak p-unc	Regions
(-40 -25 +52)	548	0.001436	0.001704	0.000014	0.999674	0.000032	476 voxels (87%) covering 2% of PostCG l (Postcentral Gyrus Left) with center at (-44,-22,+48)<br>57 voxels (10%) covering 0% of PreCG l (Precentral Gyrus Left) with center at (-34,-23,+47)<br>15 voxels (3%) covering 0% of not-labeled with center at (-32,-25,+47)
(-01 -65 +16)	495	0.002994	0.001704	0.000028	0.985124	0.000014	426 voxels (86%) covering 1% of Precuneous (Precuneous Cortex) with center at (-2,-64,+16)<br>34 voxels (7%) covering 0% of LG l (Lingual Gyrus Left) with center at (-3,-61,+7)<br>13 voxels (3%) covering 0% of ICC l (Intracalcarine Cortex Left) with center at (-2,-65,+12)<br>3 voxels (1%) covering 0% of SCC l (Supracalcarine Cortex Left) with center at (-2,-67,+15)<br>3 voxels (1%) covering 0% of Ver45 (Vermis 4 5) with center at (-1,-58,+6)

Supplementary Table 1: Group level analysis results of seed based connectivity maps

							2 voxels (0%) covering 0% of SCC r (Supracalcarine Cortex Right) with center at (+2,-66,+18)<br>1 voxels (0%) covering 0% of Cuneal r (Cuneal Cortex Right) with center at (+2,-68,+20)<br>13 voxels (3%) covering 0% of not-labeled with center at (+0,-63,+10)
(+36 -14 +63)	490	0.003213	0.001704	0.000030	0.654874	0.000003	488 voxels (100%) covering 1% of PreCG r (Precentral Gyrus Right) with center at (+35,-13,+62)<br>2 voxels (0%) covering 0% of not-labeled with center at (+35,-14,+56)
(-64 -42 +23)	418	0.009118	0.003638	0.000086	0.996663	0.000021	205 voxels (49%) covering 2% of pSMG l (Supramarginal Gyrus, posterior division Left) with center at (-63,-42,+24)<br>94 voxels (22%) covering 2% of PO l (Parietal Operculum Cortex Left) with center at (-60,-38,+23)<br>48 voxels (11%) covering 1% of aSMG l (Supramarginal Gyrus, anterior division Left) with center at (-65,-39,+27)<br>1 voxels (0%) covering 0% of PT l (Planum Temporale Left) with center at (-62,-38,+19)<br>70 voxels (17%) covering 0% of not-labeled with center at (-66,-40,+25)
