# Supplementary material for: Shared neurobiological changes in individuals with Crohn’s disease and major depressive disorder
Source: Commun Med (Lond). 2025 Sep 17;5:388. doi: 10.1038/s43856-025-01117-w (PMC12443968; doi:10.1038/s43856-025-01117-w)
Supplement: Supplementary file 4 — Supplementary Data 2 [file 43856_2025_1117_MOESM4_ESM.pdf]

Supplementary Data 2

Supplementary Table 2: Individual BDI-II scores

Healthy Controls (HC)	Crohn's Disease Patients (CD)	Major Depressive Disorder (MDD)
0	26	27
2	2	49
7	7	52
0	2	29
0	1	39
0	7	32
2	0	27
0	7	16
1	3	37
0	5	31
2	1	12
2	0	20
0	11	21
0	24	36
1	0	7
0	14	32
3	3	32
0	15	39

Supplementary Table 2 presents the individual BDI-II scores for all subjects across groups and serves as the source data for Figure 1.

Supplementary Table 3: Individual BDI-II and GSRS scores

BDI-II Score	GSRS Total Score	GSRS Reflux	GSRS Abdominal pain	GSRS Indigestion	GSRS Diarrhoea	GSRS Constipation
26	4,07	2	3,67	5,25	4,67	3,67
2	1,14	1	1,67	1	1	1
7	3,34	5	3,33	7	3,67	1,33
2	1,6	1	2	2	1,33	1,33
1	1,54	2	1,67	1,5	1	1,67
7	1,14	1	1	1,5	1	1
7	3,13	1,5	2	4,5	3,67	3
3	3,8	2,5	2,33	3,75	7	3
5	2	2,5	2,33	2,25	1,67	1,33
1	1,74	1	1,33	2,25	1,67	2
0	1,63	1	1	2,5	1	1,67
11	2,87	3,5	2	3,5	2	3,33
24	3,27	2	3,67	5	3	1,67
0	1,27	1	1,33	1,5	1,33	1
14	1,066	1	1	1	1	1,33
3	1,87	2	2	2,25	1,67	1,33
0	1,53	1	1	3	1	1
15	3,13	4	3,33	3,75	1,67	3

Supplementary Table 3 presents the individual IBD and GSRS scores for all participants in the CD group and serves as the source data for Figure 2.

Supplementary Table 4: Individual fALFF values

Healthy Controls (HC)	Crohn’s Disease Patients (CD)	Major Depressive Disorder (MDD)
2,040803218	2,242697136	0,884049503
1,409463763	0,908809876	1,071834802
2,079982668	1,342989316	2,288879867
1,764255884	0,843572643	1,13547621
1,852966061	1,360359871	1,575165425
1,661582205	1,567486245	0,785012221
1,633015348	1,178372115	0,741143561
1,535425845	1,321482054	0,499222185
1,932478565	1,578639148	1,326129787
1,839837478	1,310515802	1,873208626
1,747856622	1,055828096	1,221490733
1,528830319	1,23998681	1,561543494
1,714199567	1,95046748	2,145969152

Supplementary Table 4 presents the individual fALFF values for all participants across groups and serves as the source data for Figure 3(c).
