# Supplementary material for: Shared neurobiological changes in individuals with Crohn’s disease and major depressive disorder
Source: Commun Med (Lond). 2025 Sep 17;5:388. doi: 10.1038/s43856-025-01117-w (PMC12443968; doi:10.1038/s43856-025-01117-w)
Supplement: Supplementary file 5 — Reporting Summary [file 43856_2025_1117_MOESM5_ESM.pdf]

nature portfolio

Corresponding author(s): Ravichandran Rajkumar, Irene Neuner

Last updated by author(s): 04.07.2025

# Reporting Summary

Nature Portfolio wishes to improve the reproducibility of the work that we publish. This form provides structure for consistency and transparency in reporting. For further information on Nature Portfolio policies, see our Editorial Policies and the Editorial Policy Checklist.

## Statistics

For all statistical analyses, confirm that the following items are present in the figure legend, table legend, main text, or Methods section.

| n/a | Confirmed | |
|---|---|---|
| ☐ | ☒ | The exact sample size ($n$) for each experimental group/condition, given as a discrete number and unit of measurement |
| ☐ | ☒ | A statement on whether measurements were taken from distinct samples or whether the same sample was measured repeatedly |
| ☐ | ☒ | The statistical test(s) used AND whether they are one- or two-sided<br>*Only common tests should be described solely by name; describe more complex techniques in the Methods section.* |
| ☐ | ☒ | A description of all covariates tested |
| ☐ | ☒ | A description of any assumptions or corrections, such as tests of normality and adjustment for multiple comparisons |
| ☐ | ☒ | A full description of the statistical parameters including central tendency (e.g. means) or other basic estimates (e.g. regression coefficient) AND variation (e.g. standard deviation) or associated estimates of uncertainty (e.g. confidence intervals) |
| ☐ | ☒ | For null hypothesis testing, the test statistic (e.g. $F$, $t$, $r$) with confidence intervals, effect sizes, degrees of freedom and $P$ value noted<br>*Give P values as exact values whenever suitable.* |
| ☐ | ☐ | For Bayesian analysis, information on the choice of priors and Markov chain Monte Carlo settings |
| ☐ | ☐ | For hierarchical and complex designs, identification of the appropriate level for tests and full reporting of outcomes |
| ☐ | ☒ | Estimates of effect sizes (e.g. Cohen's $d$, Pearson's $r$), indicating how they were calculated |

*Our web collection on statistics for biologists contains articles on many of the points above.*

## Software and code

Policy information about availability of computer code

Data collection

Crohn's disease (CD) patients (n = 18, age = 29,79 ± 6,84; 12 males) along with age matched MDD patients (n = 18, age = 28,97 ± 5,96; 13 males), and healthy controls (HC) (n = 18, age = 28,35 ±, 5,23; 12 males) were included in the study. The CD patients were recruited from the Clinic for Gastroenterology, Metabolic Diseases, and Internal Intensive Care Medicine (Medical Clinic III), University Hospital Aachen, Germany. The CD patients were diagnosed based on results from colonoscopy and histology, which were evaluated by experienced gastroenterologists in the clinic. In addition to the clinical diagnosis, gastrointestinal symptoms in CD patients were assessed via the Gastrointestinal Symptom Rating Scale (GSRS), 17 licenced from AstraZeneca AB, Sweden. The GSRS questionnaire consists of 15 items, divided into five sub-scores. These sub-scores describe the symptom clusters of reflux, abdominal pain, indigestion, diarrhoea and constipation. A 7-point scale reflects the current status of the symptom severity profile, where 1 describes the absence of the symptom, and 7 is the highest symptom severity 18,19.
The MDD patients were recruited from the Clinic for Psychiatry, Psychotherapy and Psychosomatics, University Hospital Aachen. The MDD patients were diagnosed based on ICD -10 and DSM-5 criteria and an absence of psychotic features. The HC group was recruited based on having no history of neurologic or psychiatric disorders, as determined by the German version 6.0.0 of the Mini International Neuropsychiatric Interview (MINI) 20. The handedness of all the subjects was assessed using the Edinburgh Handedness Inventory. Only right-handed subjects with no contraindication for 7T MRI were included in the study. Additionally, depression symptom severity in all subjects was assessed using the German version of the Beck Depression Inventory-II (BDI-II)16. The BDI-II consists of 21 multiple-choice items, with four possible responses each.
MRI data acquisition was performed at Forschungszentrum Juelich using a 7T Magnetom Terra scanner (Siemens Healthineers, Erlangen, Germany) equipped with a 1Tx/32Rx Head Coil 7T Clinical (Nova Medical, Wilmongton, MA, USA). Resting-state fMRI data were acquired using a 2D T2* weighted multiband accelerated echo planar imaging (EPI) sequence developed at the Center for Magnetic Resonance Research (CMRR), Minneapolis, MN, USA (https://www.cmrr.umn.edu/multiband/)21–23. The entire brain was covered with a field of view (FOV) of 220 x 220 mm2, a matrix size of 168 x 168, and a slice thickness of 1.3mm. In total, 305 volumes with 100 slices each were acquired with a

repetition time (TR) of 2000 ms, an echo time (TE) of 25 ms, and a flip angle (FA) of 70° using a multiband factor of 4. Subjects were instructed to close their eyes and not to fall asleep during the resting-state measurement. In addition, the lights in the scanner room were switched off during the entire resting-state measurement. To correct for susceptibility-induced geometric distortions, two additional fMRI volumes were recorded with opposite phase encoding direction (posterior-anterior phase-encoding).
Structural images were obtained using a T1-weighted MP2RAGE. The MP2RAGE acquires two gradient echo images with different inversion times (TI) and flip angles (FA) (inversion image 1 (INV1) TI = 840 ms, flip, FA = 4°, INV2 TI= 2370 ms, FA = 5°). The other sequence-related parameters were similar for both gradient echo images: echo time (TE) = 1.99 ms; repetition time (TR) = 4500 ms for signal-to-noise ratio (SNR) optimization. The image matrix was set to 320 x 300, achieving an isotropic resolution of 0.75 mm3 in 208 sagittal slices. The T1-weighted anatomical images referred to here were produced by combining the two gradient echo images by means of a ratio 24.

Data analysis

fThe raw DICOM scans (structural and functional) were 3D converted into the neuroimaging informatics technology initiative (NIfTI) format using the dcm2niix tool . The 3D structural and functional images were visually audited to check for poor scan quality, artefacts and abnormal tissues using FSL View software (https://fsl.fmrib.ox.ac.uk/fsl/fslwiki/FslView). MRI images were pre-processed using CONN release 22.a2 SPMrelease 12.7771 and MATLAB (R2022a).
All statistical analysis was performed using the MATLAB (R2022a) software package. Differences in BDI-II scores were compared using a rank-based nonparametric Kruskal-Wallis test (kruskalwallis function in MATLAB). Subsequent post-hoc analyses were conducted using the multcompare function in MATLAB to determine the specific group differences. Within each Kruskal-Wallis test, the corrections for multiple comparisons was performed using the Bonferroni correction method with a significance level of 5%.
To explore associations between gastrointestinal symptoms and depression symptom severity in CD patients, a correlation analysis was performed between the GSRS total as well as the sub-scores and BDI-II scores, respectively. Spearman's correlation coefficients were computed with a significance level of 5%. In all of the correlation analyses, the family-wise error rate (FWER), due to multiple comparisons, was controlled for using a permutation test. 1000 permutations were performed for each comparison (correlation), and the p-value was adjusted using the "max statistics" method.

(The refences and links to the softwares are provided in the manuscript)

For manuscripts utilizing custom algorithms or software that are central to the research but not yet described in published literature, software must be made available to editors and reviewers. We strongly encourage code deposition in a community repository (e.g. GitHub). See the Nature Portfolio guidelines for submitting code & software for further information.

## Data

Policy information about availability of data

All manuscripts must include a data availability statement. This statement should provide the following information, where applicable:

- Accession codes, unique identifiers, or web links for publicly available datasets
- A description of any restrictions on data availability
- For clinical datasets or third party data, please ensure that the statement adheres to our policy

The raw MR imaging data supporting the results of this study are not openly available for ethical reasons and will be made available to the corresponding authors on reasonable request.

## Human research participants

Policy information about studies involving human research participants and Sex and Gender in Research.

Reporting on sex and gender

Sex and gender was determined based on both self-reporting and biological attributes. No sex- and gender-based analysis was conducted, as it was not relevant to the scope of our research question.

Population characteristics

Crohn's disease patients (n = 18, age = 29,79 ± 6,84; 12 males) along with age matched major depressive disorder (MDD) patients (n = 18, age = 28,97 ± 5,96; 13 males), and healthy controls (HC) (n = 18, age = 28,35 ±, 5,23; 12 males) were included in the study.

Recruitment

Participants were recruited at the University Hospital Aachen. CD patients at the Department of Gastroenterology and MDD patients at the Department of Psychiatry, Psychotherapy and Psychosomatics. Healthy controls were recruited via flyers.

Ethics oversight

Ethics Committee of the Medical Faculty of RWTH Aachen University

Note that full information on the approval of the study protocol must also be provided in the manuscript.

# Field-specific reporting

Please select the one below that is the best fit for your research. If you are not sure, read the appropriate sections before making your selection.

☒ Life sciences ☐ Behavioural & social sciences ☐ Ecological, evolutionary & environmental sciences

For a reference copy of the document with all sections, see nature.com/documents/nr-reporting-summary-flat.pdf

# Life sciences study design

All studies must disclose on these points even when the disclosure is negative.

| | |
|---|---|
| Sample size | Our sample size contains 18 subjects for each group. 18 Chrons disease patients, 18 MDD-patients and 18 Healthy controls. |
| Data exclusions | Due to poor quality fMRI scans and artefacts, data from five CD patients were excluded. |
| Replication | The results presented in this study have been subjected to rigorous statistical testing, including permutation, FWER... where appropriate. Additional reproducibility testing was not performed. |
| Randomization | The participants where allocated into groups by their clinical diagnosis. Three groups: Chron´s disease, MDD and healthy controls. |
| Blinding | Blinding was not relevant to our study, as the investigators had no impact on the outcome of the tests we applied(MRI + questionnaires). |

# Reporting for specific materials, systems and methods

We require information from authors about some types of materials, experimental systems and methods used in many studies. Here, indicate whether each material, system or method listed is relevant to your study. If you are not sure if a list item applies to your research, read the appropriate section before selecting a response.

## Materials & experimental systems

| n/a | Involved in the study | |
|---|---|---|
| ☒ | ☐ | Antibodies |
| ☒ | ☐ | Eukaryotic cell lines |
| ☒ | ☐ | Palaeontology and archaeology |
| ☒ | ☐ | Animals and other organisms |
| ☒ | ☐ | Clinical data |
| ☒ | ☐ | Dual use research of concern |

## Methods

| n/a | Involved in the study | |
|---|---|---|
| ☒ | ☐ | ChIP-seq |
| ☒ | ☐ | Flow cytometry |
| ☐ | ☒ | MRI-based neuroimaging |

# Magnetic resonance imaging

## Experimental design

| | |
|---|---|
| Design type | Resting state fMRI - 7Tesla |
| Design specifications | Eyes closed, resting fMRI measurement performed |
| Behavioral performance measures | Eyes closed, resting fMRI measurement performed |

## Acquisition

| | |
|---|---|
| Imaging type(s) | Structural and functional MRI |
| Field strength | 7 Tesla |
| Sequence & imaging parameters | Resting-state fMRI data were acquired using a 2D T2* weighted multiband accelerated echo planar imaging (EPI) sequence developed at the Center for Magnetic Resonance Research (CMRR), Minneapolis, MN, USA (https://www.cmrr.umn.edu/multiband/). In total, 305 volumes with 100 slices each were acquired with a repetition time (TR) of 2000 ms, an echo time (TE) of 25 ms, and a flip angle (FA) of 70° using a multiband factor of 4.To correct for susceptibility-induced geometric distortions, two additional fMRI volumes were recorded with opposite phase encoding direction (posterior-anterior phase-encoding). |
| Area of acquisition | Whole brain scan |

Diffusion MRI ☐ Used ☒ Not used

## Preprocessing

| | |
|---|---|
| Preprocessing software | The raw DICOM scans (structural and functional) were 3D converted into the neuroimaging informatics technology initiative (NIfTI) format using the dcm2niix tool. The susceptibility-induced off-resonance field (fieldmap) was initially estimated using a method similar to that described in 25 as implemented in FSL26. In the next step, the fMRI images were pre-processed using CONN13 release 22.a27, SPM12 release 12.7771 and MATLAB (R2022a). |

| | |
|---|---|
| Normalization | MNI-space normalization |
| Normalization template | Both functional and anatomical data were normalized to standard MNI space, segmented into grey matter, white matter, and CSF tissue classes, and resampled to 1 mm isotropic voxels following a direct normalization procedure35,36 using the SPM unified segmentation and normalization algorithm with the default IXI-549 tissue probability map template. |
| Noise and artifact removal | Functional data were also denoised using a standard denoising pipeline, which included the regression of potential confounding effects such as white matter timeseries (5 CompCor noise components), CSF timeseries (5 CompCor noise components), motion parameters and their first order derivatives (12 factors), outlier scans (below 26 factors), and linear trends (2 factors), within each functional run. This was followed by band-pass frequency filtering of the BOLD timeseries48 between 0.008 Hz and 0.09 Hz. CompCor noise components within white matter and CSF were estimated by computing the average BOLD signal as well as the largest principal components orthogonal to the BOLD average, motion parameters, and outlier scans within each subject's eroded segmentation masks. Outlier detection resulted in the removal of 0 to 26 fMRI volumes per subject (median = 0), with an average retention rate of 99% of volumes per subject (range = 91.48% to 100%). |
| Volume censoring | Potential outliers were identified using ART based on framewise displacement above 0.9 mm or global BOLD signal changes above 5 standard deviations. |

## Statistical modeling & inference

| | |
|---|---|
| Model type and settings | To explore potential shared neurobiological changes in CD and MDD patients compared to HCs, a group-level general linear model (GLM)-based analysis was performed on normalized fALFF maps. For each individual voxel, a separate GLM was estimated, with the fALFF value at this voxel as the dependent variable, and group BDI-II scores, age, and gender were set as the independent variables. The group analysis was performed to test the common differences between CD and MDD patients and HCs. The contrast was designed as [1 -0.5 -0.5 0 0 0] for HCs, CD patients, MDD patients, age, gender, and BDI-II score, respectively, to directly compare the average effect of CD and MDD patients against the HCs. Voxel-level hypotheses were evaluated using multivariate parametric statistics with random effects across subjects. Inferences were performed at the level of individual clusters (groups of contiguous voxels). Cluster-level inferences were based on parametric statistics from Gaussian Random Field theory. Results were thresholded using a combination of a cluster-forming p < 0.01 voxel-level threshold and a familywise corrected p-FDR < 0.05 cluster-size threshold.<br><br>To identify functional connectivity differences, whole-brain seed-based connectivity (SBC) maps were generated using seed regions defined by the entire significant cluster regions identified in the fALFF group comparison analysis. |
| Effect(s) tested | Group-level analysis was performed on SBC maps using separate GLM models to test differences between the HC group and the MDD group (HC > MDD), the HC group and the CD group (HC > CD), the HC group compared to a combined average of the MDD and CD groups (HC > 0.5MDD + 0.5CD), and regions with altered connectivity between the MDD group and CD group (MDD > CD)36. Within each model, a separate GLM was estimated for each individual voxel, with first-level SBC measures at this voxel as the dependent variable and the group BDI-II scores, age, and gender as the independent variables. Results were thresholded using a combination of a cluster-forming p < 0.001 voxel-level threshold and a familywise corrected p-FDR < 0.05 cluster-size threshold52. Corrections for multiple comparisons across the four separate GLM models (HC > MDD, HC > CD, HC > 0.5MDD + 0.5CD, and MDD > CD) were not performed in this analysis. |

Specify type of analysis: ☐ Whole brain ☐ ROI-based ☒ Both

| | |
|---|---|
| Anatomical location(s) | whole-brain seed-based connectivity maps (SBC) were estimated using 10 mm spherical seed regions created from the significant peak cluster positions of fALFF |
| Statistic type for inference (See Eklund et al. 2016) | SBC Results were thresholded using a combination of a cluster-forming p < 0.001 voxel-level threshold and a familywise corrected p-FDR < 0.05 cluster-size threshold |
| Correction | SBC Results were thresholded using a combination of a cluster-forming p < 0.001 voxel-level threshold and a familywise corrected p-FDR < 0.05 cluster-size threshold |

## Models & analysis

| n/a | Involved in the study |
|---|---|
| ☐ | ☒ Functional and/or effective connectivity |
| ☐ | ☐ Graph analysis |
| ☐ | ☐ Multivariate modeling or predictive analysis |

| | |
|---|---|
| Functional and/or effective connectivity | Functional connectivity strength was represented using Fisher-transformed bivariate correlation coefficients from a weighted GLM. Functional connectivity strength was defined separately for each pair of seed and target voxels, modelling the association between their BOLD signal timeseries |
| Graph analysis | Not applicable |
| Multivariate modeling and predictive analysis | Not applicable |
